# Supplementary material for: Yiai Fuzheng decoction inhibits triple-negative breast cancer by remodeling the immune microenvironment
Source: Front Immunol. 2025 Sep 30;16:1615631. doi: 10.3389/fimmu.2025.1615631 (PMC12518410; doi:10.3389/fimmu.2025.1615631)
Supplement: Supplementary file 2 [file DataSheet1.zip › Supplementary File 2/Treatment/C_vs_D/01_Basic_Statistics/Metabolite_Class_Statistics/Class_Barplot_by_Sample.pdf]

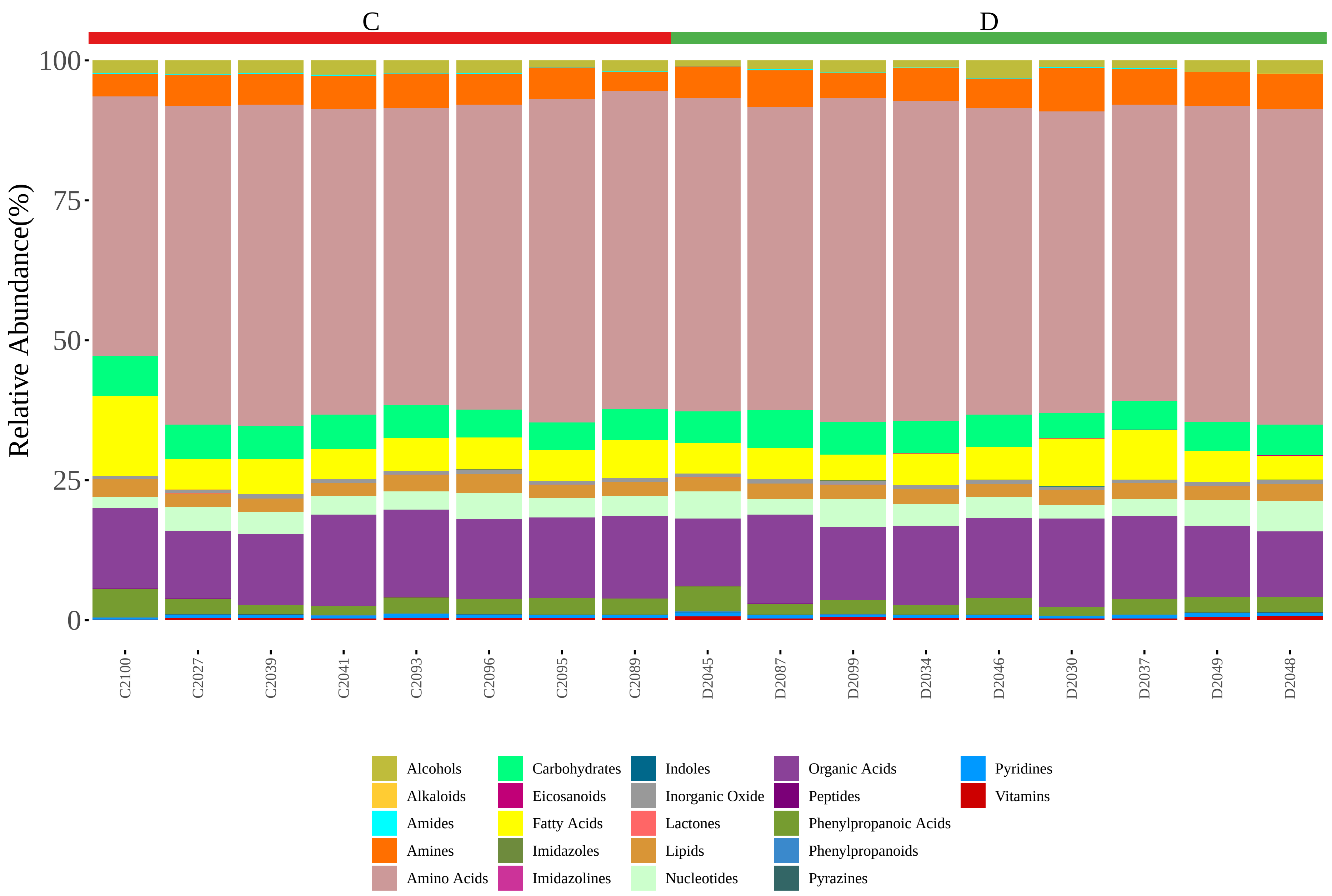
C
D
Relative Abundance(%)
100
75
50
25
0
C2100
C2027
C2039
C2041
C2093
C2096
C2095
C2089
D2045
D2087
D2099
D2034
D2046
D2030
D2037
D2049
D2048
Alcohols
Alkaloids
Amides
Amines
Amino Acids
Carbohydrates
Eicosanoids
Fatty Acids
Imidazoles
Imidazolines
Indoles
Inorganic Oxide
Lactones
Lipids
Nucleotides
Organic Acids
Peptides
Phenylpropanoic Acids
Phenylpropanoids
Pyrazines
Pyridines
Vitamins
